# PW03-013 - Behçet's disease: genotype-phenotype correlations

**DOI:** 10.1186/1546-0096-11-S1-A239

**Published:** 2013-11-08

**Authors:** M Takeuchi, T Kawagoe, A Meguro, Y Ishigatsubo, E Remmers, D Kastner, N Mizuki

**Affiliations:** 1 Department of Ophthalmology, Yokohama City University, Yokohama, Japan; 2 Inflammatory Disease Section, Medical Genetics Branch, National Human Genome Research Institute, National Institute of Health, Bethesda, USA; 3 Department of Internal Medicine and Clinical Immunology, Yokohama City University, Yokohama, Japan

## Introduction

Although the therapy for Behçet's disease (BD) has improved since infliximab was approved for refractory retinochoroiditis therapy in Japan, the exact pathogenesis of BD remains unclear. Our recent genome-wide association study has identified the *IL10* and *IL23R-IL12RB2* loci as susceptibility genes for BD, in addition to HLA-A*26 and B*51. rs1495965 is located in the intergenic region between *IL23R* and *IL12RB2* and rs1800872 is located in the promoter region of *IL10*. IL-10 is an anti-inflammatory cytokine that may have multiple effects in immunoregulation and inflammation. It is thought that regulatory T cell function related to IL-10 is an important factor in the disease pathogenesis.

## Objectives

To examine the association of *IL23R-IL12RB2* and *IL10* gene polymorphisms and HLA typing with clinical presentation of BD, and to examine the expression levels of IL10 mRNA, IL23R mRNA, and IL12RB2 mRNA from PBMCs from each genotype in healthy controls.

## Methods

A total of 464 patients with BD enrolled in our recent genome-wide association study were investigated for association between clinical manifestations and 4 susceptibility loci, rs1495965, rs1800872, HLA-A*26, and HLA-B*51. In our cohort, 196 patients had complete BD and 268 patients had incomplete BD. The expression levels of IL10 mRNA, IL23R mRNA, and IL12RB2 mRNA were examined in 33 healthy controls by real-time PCR.

## Results

The frequency of complete BD was significantly increased in patients with the risk allele of rs1800872 under genotypic and recessive models (p=0.0004, 0.0003, respectively). The frequency of skin lesions, ocular lesions, and genital ulcers was also increased in patients homozygous for the risk allele of rs1800872 (p=0.02, 0.05, 0.05, respectively). HLA-A*26, HLA-B*51, and the rs1495965 risk allele showed no association with the frequencies of complete or incomplete BD or specific clinical findings. The frequency of patients with refractory chorioretinal uveitis treated with infliximab was significantly increased among the risk allele carriers of rs1495695 and rs1800872 (p=0.01, 0.02, respectively). The expression level of IL10 mRNA was significantly decreased in the homozygotes for the rs1800872 risk allele. There were no significant differences in the expression levels of IL23R mRNA and IL12RB2 mRNA among rs1495695 risk allele carriers.

## Conclusion

This study suggests that the *IL10* polymorphism associates with complete BD and the *IL23R-IL12RB2* and *IL10* gene polymorphisms associate with the severity of uveitis. The latter association may be due to regulation of IL10 mRNA expression.

## Disclosure of interest

None declared

